# Supplementary figures and images for: Assessment of the histone mark-based epigenomic landscape in human myometrium at term pregnancy
Source: eLife. 2025 Jun 10;13:RP95897. doi: 10.7554/eLife.95897 (PMC12151536; doi:10.7554/eLife.95897)

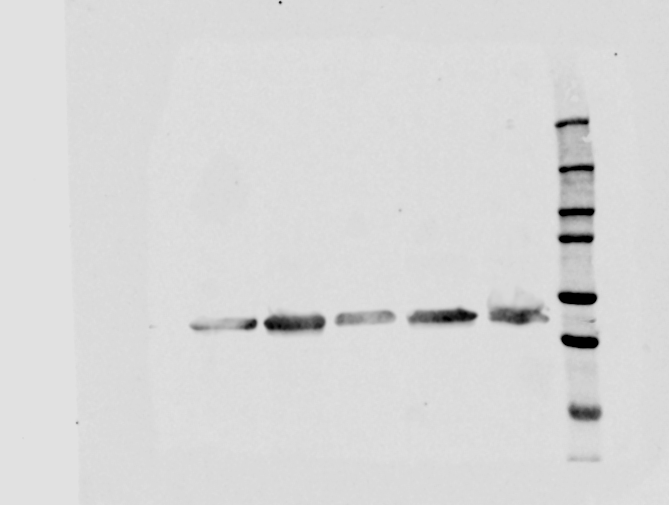

Supplement: Figure 4—source data 1. [file elife-95897-fig4-data1.zip › Source Data 1/Figure 4B-source data 1 GAPDH.tif]

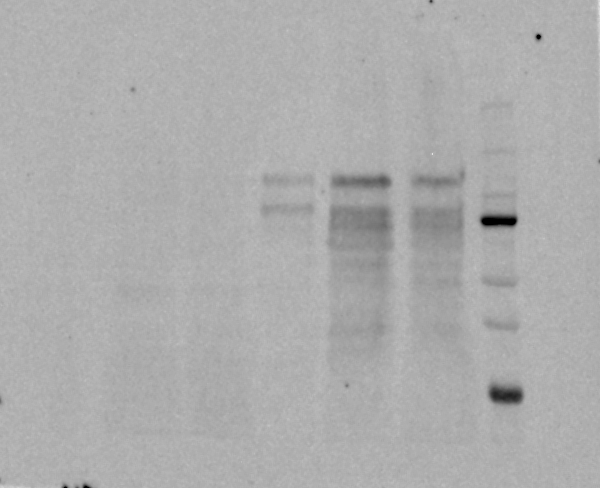

Supplement: Figure 4—source data 1. [file elife-95897-fig4-data1.zip › Source Data 1/Figure 4B-source data 1 PGR.tif]

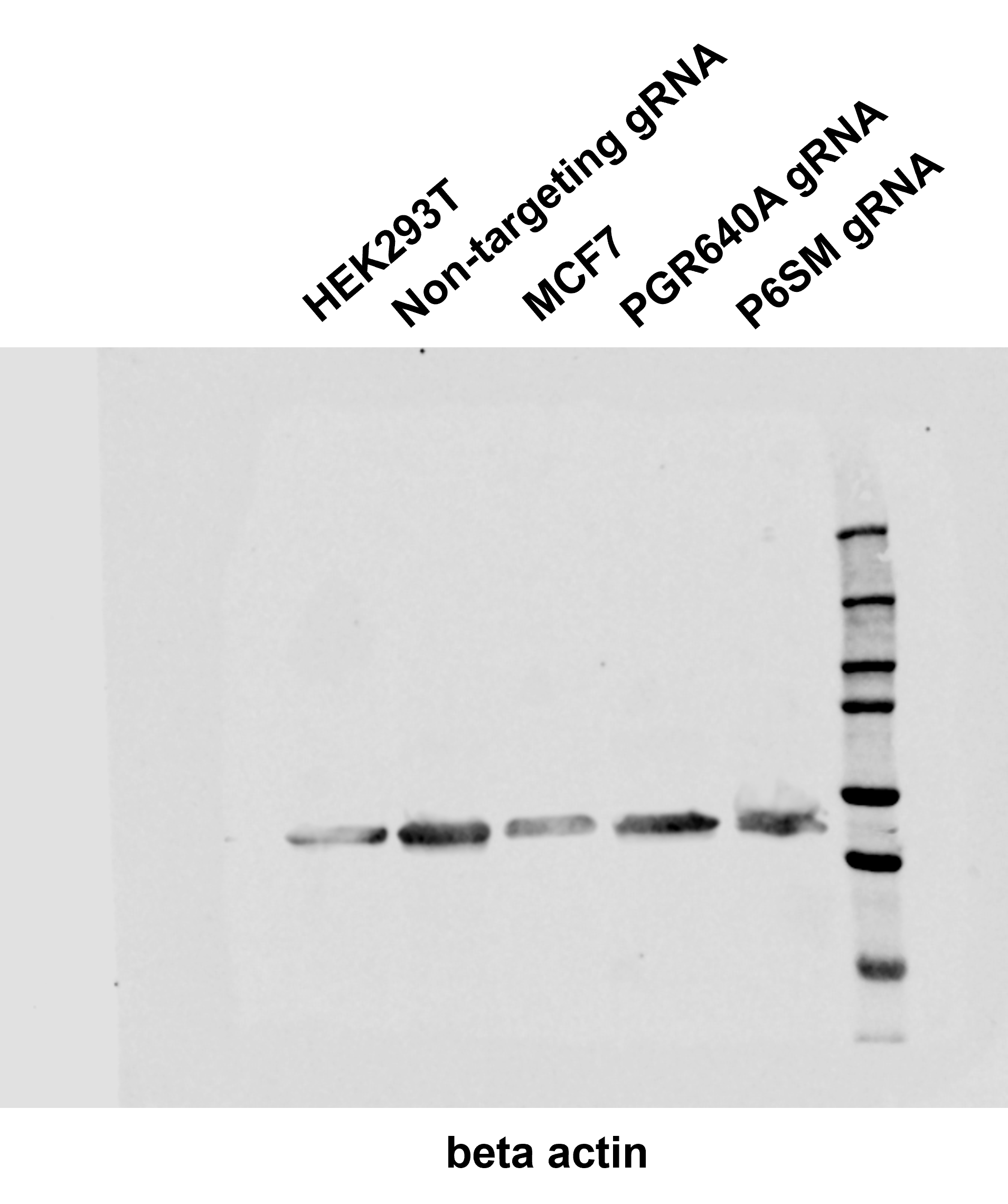

Supplement: Figure 4—source data 2. [file elife-95897-fig4-data2.zip › Source Data 2/Figure 4B-source data 2 GAPDH.png]

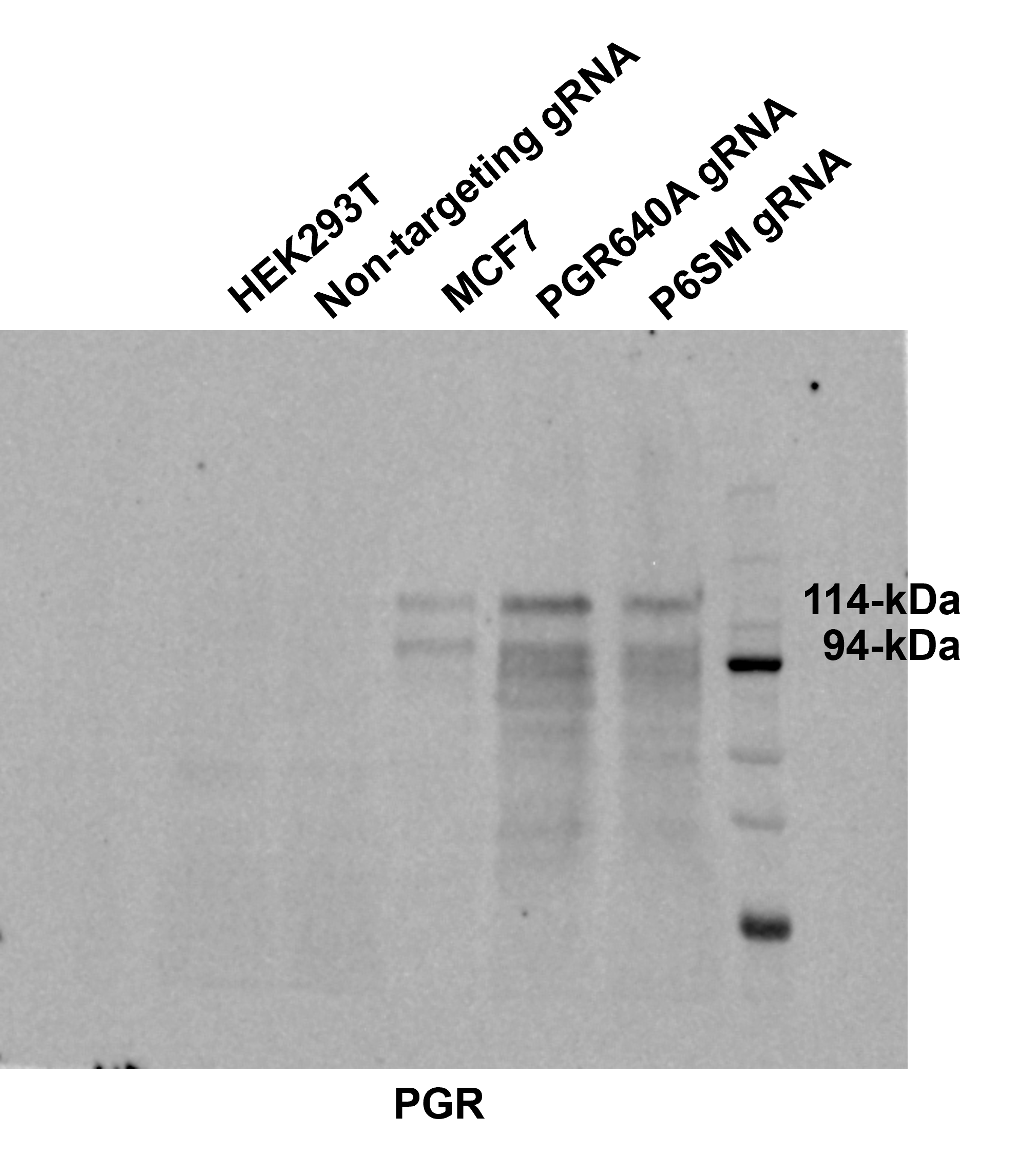

Supplement: Figure 4—source data 2. [file elife-95897-fig4-data2.zip › Source Data 2/Figure 4B-source data 2 PGR.png]
